# Paxlovid Associated with Decreased Hospitalization Rate Among Adults with COVID-19 — United States, April–September 2022

**DOI:** 10.15585/mmwr.mm7148e2

**Published:** 2022-12-02

**Authors:** Melisa M. Shah, Brendan Joyce, Ian D. Plumb, Sam Sahakian, Leora R. Feldstein, Eric Barkley, Mason Paccione, Joseph Deckert, Danessa Sandmann, Jacqueline L. Gerhart, Melissa Briggs Hagen

**Affiliations:** ^1^Coronavirus and Other Respiratory Viruses Division (proposed), National Center for Immunization and Respiratory Diseases, CDC; ^2^Epic Research, Epic Systems Corporation, Verona, Wisconsin.

Nirmatrelvir-ritonavir (Paxlovid), an oral antiviral treatment, is authorized for adults with mild-to-moderate COVID-19 who are at increased risk for progression to severe illness. However, real-world evidence on the benefit of Paxlovid, according to vaccination status, age group, and underlying health conditions, is limited. To examine the benefit of Paxlovid in adults aged ≥18 years in the United States, a large electronic health record (EHR) data set (Cosmos^†^) was analyzed to assess the association between receiving a prescription for Paxlovid and hospitalization with a COVID-19 diagnosis in the ensuing 30 days. A Cox proportional hazards model was used to estimate this association, adjusted for demographic characteristics, geographic location, vaccination, previous infection, and number of underlying health conditions. Among 699,848 adults aged ≥18 years eligible for Paxlovid during April–August 2022, 28.4% received a Paxlovid prescription within 5 days of COVID-19 diagnosis. Being prescribed Paxlovid was associated with a lower hospitalization rate among the overall study population (adjusted hazard ratio [aHR] = 0.49), among those who had received ≥3 mRNA COVID-19 vaccines (aHR = 0.50), and across age groups (18–49 years: aHR = 0.59; 50–64 years: aHR = 0.40; and ≥65 years: aHR = 0.53). Paxlovid should be prescribed to eligible adults to reduce the risk of COVID-19–associated hospitalization.

Paxlovid is an oral antiviral medication that received Emergency Use Authorization by the Food and Drug Administration on December 22, 2021 (*1*), for use in patients with mild-to-moderate COVID-19 at high risk for progression to severe illness. Eligibility for Paxlovid includes 1) receipt of a positive SARS-CoV-2 test result (including home antigen test), 2) symptoms consistent with mild-to-moderate COVID-19, 3) symptom onset within the past 5 days, 4) age ≥18 years (or age ≥12 years and weight ≥40 kg), 5) one or more risk factors for progression to severe COVID-19, 6) no known or suspected severe renal or hepatic impairment, 7) no history of clinically significant reactions (e.g., toxic epidermal necrolysis or Stevens-Johnson syndrome) to the active ingredients (nirmatrelvir or ritonavir) or other components of the product, and 8) no contraindicated medications.^§^

A retrospective analysis was performed on patient records included in Cosmos, a data set that includes EHR information from >160 million persons in U.S. health systems covered by Epic, a health care software company (https://cosmos.epic.com). Inclusion criteria comprised 1) diagnosis of COVID-19 or a positive SARS-CoV-2 test result during April 1–August 31, 2022^¶^; 2) an outpatient encounter (telemedicine, in-person, urgent care, emergency department, or other)** associated with the COVID-19 diagnosis; 3) at least one previous face-to-face encounter in Cosmos during the 3 years preceding the COVID-19 diagnosis^††^; 4) age ≥50 years, or ≥18 years with a documented underlying health condition based on *International Classification of Diseases*, *Tenth Revision* (ICD-10) codes or medical record fields^§§^; 5) not known to be pregnant; and 6) not known to have pharmacologic or medical contraindications to Paxlovid use.^¶¶^ For patients with multiple SARS-CoV-2 infections during the study period, only data from the first infection were used in the analysis; date of diagnosis (earliest COVID-19 diagnosis code or positive SARS-CoV-2 test result) was used as a proxy for symptom onset, and Paxlovid receipt was defined as receiving a prescription for Paxlovid during the 5 days after COVID-19 diagnosis.*** The primary outcome was overnight COVID-19 hospitalization during the 30 days after the date of diagnosis; secondary outcomes were all-cause hospitalization and acute respiratory illness (ARI)–associated hospitalization.^†††^

Association between Paxlovid receipt and subsequent hospitalization was assessed using a Cox proportional hazards model, including age group, sex, race and ethnicity, social vulnerability index,^§§§^ number of underlying health conditions, U.S. Census Bureau region of residence, previous COVID-19 infection, and COVID-19 vaccination status.^¶¶¶^ In-hospital COVID-19 mortality during an admission commencing during the 30-day follow-up period was described but not used as an analytic outcome because of concern about underascertainment. Persons receiving Paxlovid contributed unexposed time until the prescription date and exposed time after the prescription date; those not receiving Paxlovid contributed unexposed time. Follow-up time ended when a hospitalization occurred or at 30 days after diagnosis, whichever came first. To assess possible bias related to symptom severity at diagnosis, primary analyses were repeated either excluding telemedicine visits, or excluding patients hospitalized during the 2 days after diagnosis. This activity was reviewed by CDC and was conducted consistent with applicable federal law and CDC policy.****

Among 1,713,120 persons aged ≥18 years with a COVID-19 diagnosis during April 1–August 31, 2022, 699,848 (40.9%) met the inclusion criteria, including 198,927 who received Paxlovid within 5 days after diagnosis and 500,921 who did not (Figure). Among all persons with COVID-19 who were eligible for Paxlovid, 15.0% had documentation of previous infection and 68.8% were confirmed to have received ≥2 COVID-19 mRNA vaccine doses. Overall, 28.4% of eligible persons received Paxlovid. Paxlovid recipients were more likely to have a telehealth encounter (49.1%) than nonrecipients (18.4%, standardized mean difference = 0.69). Prevalences of underlying health conditions were similar among Paxlovid recipients and nonrecipients (Table 1), and 92.4% had at least one underlying condition. Persons who were immunocompromised^††††^ accounted for 9.4% (64,911) of the study population, 30.2% of whom received Paxlovid. During the 30 days after a COVID-19 diagnosis, 5,229 (0.75%) persons were hospitalized; 3,311 (63.3%) of these hospitalizations occurred among persons aged ≥65 years. Among the 198,927 Paxlovid recipients, 930 (0.47%) were hospitalized,^§§§§^ compared with 4,299 (0.86%) of nonrecipients. Among the 5,229 persons with a COVID-19 hospitalization, 930 (17.8%) received Paxlovid during the 5 days after diagnosis. Overall, 211 deaths were reported during a COVID-19 hospitalization. Among those who received Paxlovid, 0.01% (29 of 198,927) died compared with 0.04% (182 of 500,921) of persons who did not receive Paxlovid.

**FIGURE:**
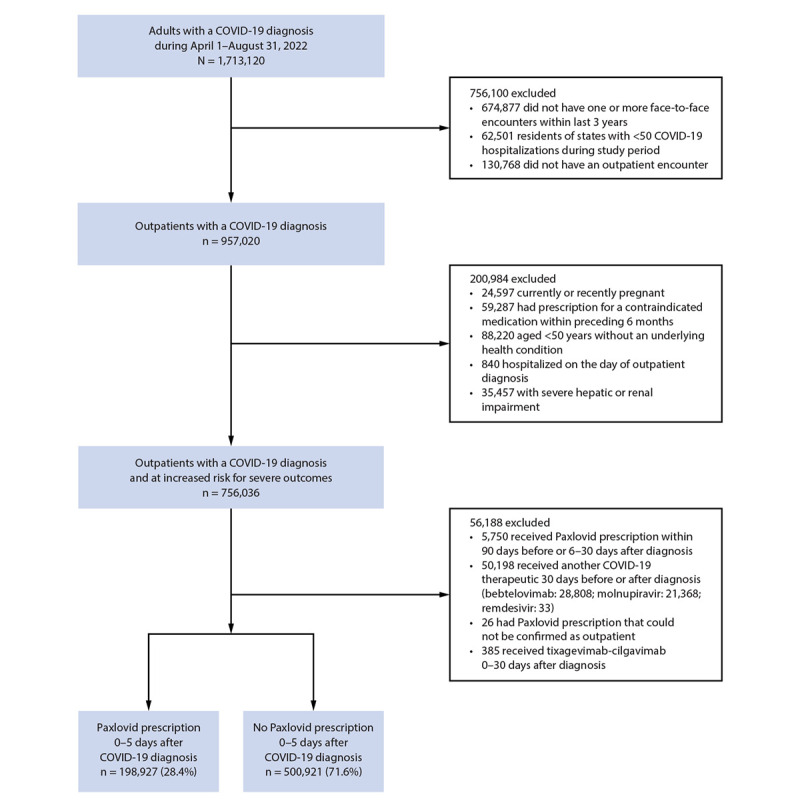
Identification of patients with COVID-19* who were eligible for treatment with Paxlovid (nirmatrelvir-ritonavir) — Cosmos,^†^ United States, April–September 2022 **Abbreviation: **NAAT = nucleic acid amplification test. * Patients were classified as having COVID-19 based on a diagnosis code for COVID-19 or based on a positive SARS-CoV-2 antigen or nucleic acid amplification test. Among 1,713,120 adults aged ≥18 years who met this definition during April 1–August 1, 2022, 930,847 had a diagnosis code only, 159,878 had a positive NAAT result only, 12,874 had a positive antigen test result only, and 609,521 had both a diagnosis code and positive test result (NAAT or antigen test). Exclusions summarized at each level of the flow chart are not mutually exclusive. ^† ^Cosmos is an electronic health record dataset that includes information from >160 million persons in U.S. health systems covered by Epic. https://cosmos.epic.com

**TABLE 1:** Characteristics of persons eligible for Paxlovid (nirmatrelvir-ritonavir) by prescription receipt within 5 days after COVID-19 diagnosis — Cosmos,* United States, April–September 2022

Characteristic	No. (column %)		Standardized mean difference
	Paxlovid prescribed (n = 198,927)	Paxlovid not prescribed (n = 500,921)	
**Age group, yrs**			
18–35	20,543 (10.3)	113,716 (22.7)	−0.34
36–49	36,077 (18.1)	107,373 (21.4)	−0.08
50–64	66,929 (33.7)	147,274 (29.4)	0.09
≥65	75,378 (37.9)	132,558 (26.5)	0.25
**Sex**			
Female	122,921 (61.8)	316,677 (63.2)	−0.03
Male	75,984 (38.2)	184,184 (36.8)	0.03
**Race and ethnicity**			
Black or African American, non-Hispanic	17,141 (8.6)	66,574 (13.3)	−0.15
Hispanic or Latino	12,088 (6.1)	38,487 (7.7)	−0.06
White, non-Hispanic	158,696 (79.8)	368,109 (73.5)	0.15
Other, non-Hispanic**^†^**	11,002 (5.5)	27,751 (5.5)	0.00
**Social vulnerability index^§^**			
0–0.25 (least vulnerable)	58,144 (29.5)	117,590 (23.7)	0.13
0.25–0.50	52,659 (26.7)	124,118 (25.0)	0.04
0.50–0.75	47,755 (24.2)	127,366 (25.7)	−0.03
0.75–1.00 (most vulnerable)	38,902 (19.7)	126,632 (25.6)	−0.14
**U.S. Census Bureau region^¶^**			
Northeast	47,737 (24.0)	134,818 (26.9)	−0.07
Midwest	78,925 (39.7)	189,000 (37.7)	0.04
South	51,784 (26.0)	140,818 (28.1)	−0.05
West	20,481 (10.3)	36,285 (7.2)	0.11
Outpatient encounter type**			
Telemedicine	97,644 (49.1)	91,916 (18.4)	0.69
In-person	56,793 (28.6)	245,004 (48.9)	−0.43
Urgent care	1,814 (0.9)	9,094 (1.8)	−0.08
Emergency department	19,872 (10.0)	98,359 (19.6)	−0.27
Other	22,804 (11.5)	56,548 (11.3)	0.01
**Underlying health conditions^††^**			
0	16,159 (8.1)	37,072 (7.4)	0.03
1	49,848 (25.1)	152,179 (30.4)	−0.12
≥2	132,920 (66.8)	311,670 (62.2)	0.10
**Immunocompromised^§§^**			
No	179,321 (90.1)	455,616 (91.0)	−0.03
Yes	19,606 (9.9)	45,305 (9.0)	0.03
**Previous infection^¶¶^**			
No	180,373 (90.7)	414,440 (82.7)	0.24
Yes	18,554 (9.3)	86,481 (17.3)	−0.24
**Obesity**			
No	100,035 (50.3)	257,590 (51.4)	−0.02
Yes	98,892 (49.7)	243,331 (48.6)	0.02
**Smoker (current or former)**			
No	119,770 (60.2)	287,747 (57.4)	0.06
Yes	79,157 (39.8)	213,174 (42.6)	−0.06
**Diabetes**			
No	161,177 (81.0)	424,246 (84.7)	−0.10
Yes	37,750 (19.0)	76,675 (15.3)	0.10
COVID-19 vaccination status***			
≥3 mRNA doses	119,324 (60.0)	209,614 (41.9)	0.37
2 mRNA doses	36,924 (18.6)	115,444 (23.1)	−0.11
Unvaccinated	30,619 (15.4)	141,931 (28.3)	−0.32
Other	12,060 (6.1)	33,932 (6.8)	−0.03
**Month of COVID-19 diagnosis**			
Apr 2022	10,581 (5.3)	50,116 (10.0)	−0.18
May 2022	36,326 (18.3)	104,105 (20.8)	−0.06
Jun 2022	40,747 (20.5)	104,418 (20.9)	−0.01
Jul 2022	58,961 (29.6)	126,991 (25.4)	0.10
Aug 2022	52,312 (26.3)	115,291 (23.0)	0.08

* Cosmos is an electronic health record dataset that includes information from >160 million persons in U.S. health systems covered by Epic. https://cosmos.epic.com

^†^ Includes American Indian or Alaska Native, Native Hawaiian or other Pacific Islander, or Asian, or other race.

^§^ https://www.atsdr.cdc.gov/placeandhealth/svi/index.html

**^¶^** https://www2.census.gov/geo/pdfs/maps-data/maps/reference/us_regdiv.pdf

^**^ Telemedicine included virtual, electronic, and telephone encounters. In-person included in-person outpatient encounters not in the urgent care or emergency department setting. Other included all other outpatient encounters which could not be categorized clearly.

^†† ^https://www.cdc.gov/coronavirus/2019-ncov/hcp/clinical-care/underlyingconditions.html (Accessed October 24, 2022).

^§§^ Immunocompromised status was defined using *International Classification of Diseases, Tenth Revision* codes (adapted from https://academic.oup.com/cid/article/73/11/e4353/6060064 or immunocompromising medication prescribed in the past 6 months (adapted from https://www.atsjournals.org/doi/10.1513/AnnalsATS.201507-415BC).

**^¶¶^** Previous infection was defined as a COVID-19 diagnosis code or positive COVID-19 nucleic acid amplification test result or antigen test result >90 days before the current diagnosis.

*** Vaccination categories included 1) unvaccinated if no COVID-19 vaccine had been received; 2) 2 mRNA dose-recipients if ≥14 days had elapsed after the second dose and no subsequent doses had been received or <7 days since receipt of third dose; 3) ≥3 mRNA dose-recipients if ≥7 days had elapsed since receipt of the third dose; and 4) other recipient if any Janssen (Johnson & Johnson) vaccine, other vaccine, or 1 mRNA vaccine dose had been received any time before COVID-19 diagnosis.

Paxlovid receipt was associated with protection against hospitalization overall (aHR = 0.49, 95% CI = 0.46–0.53) (Table 2), including among persons who had received ≥3 mRNA vaccine doses (0.50, 95% CI = 0.45–0.55) and 2 previous mRNA vaccine doses (0.50, 95% CI = 0.42–0.58). Paxlovid receipt was associated with lower hospitalization rates among persons aged 18–49 years (aHR = 0.59, 95% CI = 0.48–0.71), 50–64 years (0.40, 95% CI = 0.34–0.48), and ≥65 years (0.53, 95% CI = 0.48–0.58). Among persons aged 18–49 years, Paxlovid receipt was associated with lower hospitalization rates among persons who had received ≥3 mRNA vaccine doses (aHR = 0.75, 95% CI = 0.53–1.06) and those with only one underlying health condition (aHR = 0.91, 95% CI = 0.58–1.44), but these estimates did not reach statistical significance. Estimated protection by Paxlovid was similar by month of diagnosis. Findings from sensitivity analyses, excluding telemedicine encounters and patients hospitalized during the first 2 days after diagnosis, also indicated significant reduction in hospitalization among Paxlovid recipients.^¶¶¶¶^ In the analysis of secondary outcomes, among the overall study population, Paxlovid receipt was associated with a lower rate of all-cause hospitalization (aHR = 0.45, 95% CI = 0.43–0.48) and ARI-associated hospitalization (aHR = 0.48, 95% CI = 0.45–0.51).

**TABLE 2:** Adjusted hazard ratios for COVID-19–associated hospitalization based on Paxlovid prescription receipt (exposure) — Cosmos,* United States, April–September 2022

Characteristic	Adjusted HR (95% CI)^† ^	No. of participants	No. hospitalized	Events per 100,000 person-days		
				Overall	Exposed^§^	Unexposed^§^
**Total**	**0.49 (0.46–0.53)**	**693,084**	**5,229**	**25.31**	**15.88**	**29.05**
**COVID-19 vaccination status^¶^**						
Vaccinated (≥3 mRNA doses)	**0.50 (0.45–0.55)**	310,196	2,126	**22.98**	14.30	27.87
Vaccinated (2 mRNA doses)	**0.50 (0.42–0.58)**	149,498	1,086	**24.37**	16.37	26.92
Unvaccinated	**0.50 (0.43–0.59)**	170,789	1,477	**29.05**	19.60	31.08
UHC**						
0	**0.89 (0.58–1.36)**	52,592	106	**6.73**	6.51	6.83
1	**0.57 (0.45–0.71)**	200,116	503	**8.40**	6.46	9.03
≥2	**0.47 (0.44–0.51)**	440,376	4,620	**35.29**	20.56	41.57
**Previous infection** ^††^						
No	**0.48 (0.44–0.51)**	589,147	4,715	**26.86**	16.12	31.53
Yes	**0.76 (0.60–0.98)**	103,937	514	**16.56**	13.54	17.20
**Immunocompromised^§§^**						
No	**0.49 (0.45–0.53)**	628,706	3,770	**20.09**	12.61	23.03
Yes	**0.50 (0.44–0.58)**	64,378	1,459	**77.01**	45.99	90.49
**Month of COVID-19 diagnosis**						
Apr 2022	**0.54 (0.40–0.71)**	60,001	450	**25.16**	17.77	26.71
May 2022	**0.57 (0.48–0.67)**	139,062	979	**23.61**	17.06	25.88
Jun 2022	**0.51 (0.43–0.60)**	143,706	1,006	**23.48**	15.02	26.76
Jul 2022	**0.46 (0.40–0.53)**	184,153	1,432	**26.09**	15.65	30.94
Aug 2022	**0.44 (0.38–0.51)**	166,162	1,362	**27.52**	15.60	32.93
**Age group, yrs**						
18–49	**0.59 (0.48–0.71)**	275,930	886	**10.73**	6.99	11.68
50–64	**0.40 (0.34–0.48)**	211,940	1,032	**16.30**	7.90	20.10
≥65	**0.53 (0.48–0.58)**	205,214	3,311	**54.56**	29.72	68.80
**By age group, yrs**						
**18–49**						
Vaccinated (≥3 mRNA doses)	**0.75 (0.53–1.06)**	84,054	178	**7.07**	6.10	7.46
Vaccinated (2 mRNA doses)	**0.53 (0.35–0.82)**	70,159	198	**9.43**	6.20	10.16
Unvaccinated	**0.54 (0.39–0.76)**	97,637	417	**14.29**	9.09	15.13
1 UHC	**0.91 (0.58–1.44)**	109,620	157	**4.78**	4.11	4.91
≥2 UHC	**0.54 (0.43–0.67)**	166,310	729	**14.67**	8.35	16.54
**50–64**						
Vaccinated (≥3 mRNA doses)	**0.41 (0.30–0.55)**	98,699	284	**9.61**	5.28	12.11
Vaccinated (2 mRNA doses)	**0.46 (0.33–0.63)**	47,111	265	**18.84**	10.96	21.89
Unvaccinated	**0.38 (0.27–0.53)**	45,154	355	**26.39**	12.43	30.35
No UHC	**1.11 (0.46–2.68)**	32,519	25	**2.56**	2.87	2.46
1 UHC	**0.30 (0.17–0.55)**	53,493	109	**6.80**	2.45	8.72
≥2 UHC	**0.40 (0.33–0.48)**	125,928	898	**23.91**	11.04	30.26
**≥65**						
Vaccinated (≥3 mRNA doses)	**0.51 (0.46–0.57)**	127,443	1,664	**44.02**	24.51	57.35
Vaccinated (2 mRNA doses)	**0.53 (0.43–0.65)**	32,228	623	**65.58**	36.83	78.59
Unvaccinated	**0.58 (0.47–0.72)**	27,998	705	**85.92**	52.75	96.15
No UHC	**0.84 (0.51–1.36)**	20,073	81	**13.50**	10.34	15.49
1 UHC	**0.63 (0.47–0.85)**	37,003	237	**21.47**	13.66	26.77
≥2 UHC	**0.51 (0.47–0.56)**	148,138	2,993	**68.58**	37.33	85.48

**Abbreviations:** HR = hazard ratio; UHC = underlying health condition.

* Cosmos is an electronic health record dataset that includes information from >160 million persons in U.S. health systems covered by Epic. https://cosmos.epic.com

^† ^95% CIs that exclude 1 were considered to be statistically significant. Multivariable models were adjusted for age, sex, race and ethnicity, social vulnerability index, number of underlying health conditions, U.S. Census Bureau region of residence, previous infection, and COVID-19 vaccination status, excluding the stratum of interest.

^§^ Persons receiving Paxlovid contributed unexposed time until the prescription date and exposed time after the prescription date; those not receiving Paxlovid contributed unexposed time. Follow-up time ended when a hospitalization occurred or at 30-days after diagnosis, whichever came first.

^¶ ^Vaccination categories included 1) unvaccinated if no COVID-19 vaccine had been received; 2) 2 mRNA-dose recipients if ≥14 days had elapsed after the second dose and no subsequent doses had been received or <7 days since receipt of third dose; 3) ≥3 mRNA-dose recipients if ≥7 days had elapsed since receipt of the third dose; and 4) other recipient if any Janssen (Johnson & Johnson) vaccine, other vaccine, or 1 mRNA vaccine dose had been received any time before COVID-19 diagnosis.

** https://www.cdc.gov/coronavirus/2019-ncov/hcp/clinical-care/underlyingconditions.html (Accessed October 24, 2022).

^††^ Previous infection was defined as a COVID-19 diagnosis code or positive COVID-19 nucleic acid amplification test result or antigen test result >90 days before the current diagnosis.

^§§ ^Immunocompromised status was defined using *International Classification of Diseases, Tenth Revision* codes (adapted from https://academic.oup.com/cid/article/73/11/e4353/6060064 or immunocompromising medication prescribed during the past 6 months (adapted from https://www.atsjournals.org/doi/10.1513/AnnalsATS.201507-415BC).

## Discussion

In a sample of U.S. COVID-19 patients, many of whom had previous SARS-CoV-2 infection or were vaccinated against COVID-19, the overall COVID-19 hospitalization rate was 51% lower among those who had received a prescription for Paxlovid for presumed mild-to-moderate COVID-19, compared with those who did not. Similar benefit was seen among persons who had received ≥2 COVID-19 mRNA vaccine doses. The initial randomized clinical trial of Paxlovid, which showed an 89% reduction in severe COVID-19 outcomes, was conducted in unvaccinated persons with no previous infection during the period preceding Omicron variant predominance (*2*). This real-word analysis demonstrated that being prescribed Paxlovid is associated with a substantially reduced hospitalization risk among persons with previous immunity from infection or vaccination in the setting of the current circulating Omicron subvariants. These findings parallel those of other studies indicating added protection from Paxlovid even among persons with previous infection or vaccination (*3*–*8*). Paxlovid conferred stable protection during a period in which multiple Omicron subvariants predominated in the United States. Protection against different predominant SARS-CoV-2 subvariants is consistent with Paxlovid’s mechanism of action, which inhibits a highly conserved viral protease (*9*).

 Current guidelines for Paxlovid indicate that persons who are at high risk for progression to severe COVID-19–associated outcomes should be considered for Paxlovid, with older age being a predominant risk factor (*10*). A study from Israel among persons with mild-to-moderate COVID-19 found comparable benefit from Paxlovid against severe outcomes among persons aged ≥65 years but did not find statistical evidence of protection among younger age groups (*3*). The current analysis adds to overall evidence of protection from Paxlovid by finding a statistically significant benefit among adults aged 18–64 years, specifically among adults aged 50–64 years with one or more underlying health condition and those aged 18–49 years with two or more underlying health conditions. Although ascertainment of deaths was limited to those with a documented death during the COVID-19 hospital admission, the proportion of persons with in-hospital death was also lower among persons who received Paxlovid (0.01%) than among those who did not (0.04%).

The findings in this report are subject to at least seven limitations. First, receipt of a Paxlovid prescription is a proxy for use of Paxlovid. Paxlovid course completion could not be confirmed, which might bias the results toward the null. Second, dates of diagnosis or test positivity were used to estimate illness onset but might not reflect date of symptom onset, or the presence of mild-to-moderate COVID-19 symptoms. Third, possible inclusion of asymptomatic COVID-19 infection in the nonrecipient comparison group could bias estimates toward the null. Fourth, participants with mild illness might be overrepresented among Paxlovid prescription recipients compared with nonrecipients, given the higher proportion of telemedicine visits, potentially leading to overestimation of protection from Paxlovid; however, a sensitivity analysis restricted to in-person encounters showed similar overall results. Fifth, underlying health conditions and immunocompromise were approximated using ICD-10 codes or medical record fields and might not capture the exact prevalences of these conditions. Sixth, although available vaccination information is automatically collected at each encounter, incomplete information could have limited differences in estimates by vaccination status. Finally, hospitalizations might be incompletely ascertained in Cosmos; this limitation was mitigated by including only persons with previous face-to-face encounters, indicating higher likelihood of hospitalization within a participating health system.

This study demonstrates that Paxlovid provides protection against severe COVID-19–associated outcomes among persons for whom it is recommended, including those with vaccine-conferred immunity, and that it is underutilized among eligible persons with COVID-19. In this analysis, only 28% of eligible persons were prescribed Paxlovid. The ease of oral administration, short duration of therapy, and lower likelihood for resistance make Paxlovid a useful antiviral. Reduction in nonsevere outcomes, such as duration, number, and intensity of COVID-19 symptoms, requires further study. Paxlovid should be offered to eligible persons to protect against COVID-19 hospitalizations, irrespective of vaccination status, and especially among groups with the highest risk for severe outcomes, such as older adults and those with multiple underlying health conditions.

**Summary**

**What is already known about this topic?**

Nirmatrelvir-ritonavir (Paxlovid) is an outpatient antiviral medication recommended for adults with mild-to-moderate COVID-19 who have elevated risk of severe illness.

**What is added by this report?**

Among U.S. adults diagnosed with COVID-19, including those with previous infection or vaccination, persons who were prescribed Paxlovid within 5 days of diagnosis had a 51% lower hospitalization rate within 30 days after diagnosis than those who were not prescribed Paxlovid.

**What are the implications for public health practice?**

Paxlovid should be offered to eligible adults irrespective of vaccination status, especially in groups with the highest risk for severe COVID-19 outcomes, such as older adults and those with multiple underlying health conditions.

## Footnotes

* These authors contributed equally to this report.

†
https://cosmos.epic.com/

§
https://www.fda.gov/media/158165/download

¶ ICD-10 codes U07.1, J12.81, J12.82 and Systematized Nomenclature of Medicine–Clinical Terms (SNOMED-CT) code 840539006. Positive SARS-CoV-2 test results could be from a nucleic acid amplification test (NAAT) or an antigen test.

** Telemedicine included virtual, electronic, and telephone encounters. In-person included in-person outpatient encounters not in the urgent care or emergency department setting. Other included all other outpatient encounters which could not be categorized clearly.

†† A previous documented face-to-face encounter suggests a person’s familiarity with and ability to access care in this health system, which was used to increase the likelihood that subsequent hospitalizations were captured.

§§ Underlying health conditions were identified using ICD-10 codes, with two exceptions, obesity and smoking, which were identified using dedicated EHR fields. Persons aged <50 years were required to have at least one underlying health condition to be considered eligible for Paxlovid in this study. https://www.cdc.gov/coronavirus/2019-ncov/hcp/clinical-care/underlyingconditions.html (Accessed October 24, 2022).

¶¶ Persons with ICD-10 codes consistent with Child-Pugh Class C (indicating advanced hepatic dysfunction) or estimated glomerular filtration rate <30 mL/minute within the past 6 months were considered ineligible to receive Paxlovid. Medications contraindicated with Paxlovid were taken from Food and Drug Administration’s Fact Sheet for Healthcare Providers: Emergency Use Authorization for Paxlovid. https://www.fda.gov/media/155050/download

*** Persons were excluded from the analysis if Paxlovid was prescribed within the 90 days preceding the diagnosis date for the present episode (because of concerns about not capturing the actual COVID-19 diagnosis date), if Paxlovid was prescribed 6–30 days after diagnosis date, or if the patient received other COVID-19–directed therapeutic agents prior to hospitalization.

††† COVID-19 hospitalization was defined as having a COVID-19–specific diagnosis code (ICD-10 U07.1 or SNOMED-CT 840539006) associated with the admission. ARI-associated hospitalizations were defined using ICD-10 codes (adapted from https://doi.org/10.1056/NEJMoa2110362).

§§§
https://www.atsdr.cdc.gov/placeandhealth/svi/index.html

¶¶¶ Previous infection was defined as a COVID-19 diagnosis code or positive SARS-CoV-2 test result (NAAT or antigen) >90 days earlier. Vaccination categories included 1) unvaccinated if no COVID-19 vaccine had been received; 2) 2 mRNA-dose recipients if ≥14 days had elapsed since receipt of the second dose and no subsequent doses had been received or <7 days since receipt of third dose; 3) ≥3 mRNA-dose recipients if ≥7 days had elapsed since receipt of the third dose; and 4) other recipient if any Janssen (Johnson & Johnson) vaccine, other vaccine, or only 1 mRNA vaccine dose had been received any time before COVID-19 diagnosis. The proportional hazards assumption was evaluated by plotting hazard functions for each variable in the model.

**** 45 C.F.R. part 46, 21 C.F.R. part 56; 42 U.S.C. Sect. 241(d); 5 U.S.C. Sect. 552a; 44 U.S.C. Sect. 3501 et seq.

†††† Immunocompromise was defined using ICD-10 codes (adapted from https://academic.oup.com/cid/article/73/11/e4353/6060064) or immunocompromising medication (adapted from https://www.atsjournals.org/doi/10.1513/AnnalsATS.201507-415BC) prescribed during the past 6 months.

§§§§ COVID-19 hospitalizations occurred a median of 3 days (range = 1–30 days) after diagnosis. For those prescribed Paxlovid who were subsequently hospitalized, hospitalization occurred a median of 5 days after the Paxlovid prescription (range = 1–30 days).

¶¶¶¶ In a sensitivity analysis limited to in-person encounters at the time of diagnosis, aHR was 0.53 (95% CI = 0.48–0.58). In a second sensitivity analysis excluding persons hospitalized during the first 2 days after diagnosis, aHR was 0.63 (95% CI = 0.58–0.69).
